# Supplementary material for: Electroconvulsive shock and transcranial magnetic stimulation do not alter the survival or spine density of adult-born striatal neurons
Source: PLoS One. 2025 Jan 28;20(1):e0316717. doi: 10.1371/journal.pone.0316717 (PMC11774372; doi:10.1371/journal.pone.0316717)
Supplement: Attachment — Submitted filename: Response to Reviews.docx [file pone.0316717.s002.docx]

**Please find below our responses to the reviewers’ comments and suggestions, in bolded text. We have made many changes to the revised manuscript (highlighted in yellow in the marked-up version), which we feel significantly improves the quality of the report.**

Reviewer #1: Gaertner et al have investigated the effect of two neurostimulation techniques on the survival or adult born striatal neurons. The study specifically studies the effect of the stimulation on neurons born prior to the stimulation regime. Although the study has some small sample sizes with some animals only have 1 neuron counted for dendritic spine density, it is well designed and well written. Publishing negative results is critical and informative and the authors should be congratulated on publishing these negative findings.

I only have minor corrections that should be easy to address.

Methods:
- please provide an estimate of the electric field induced or at least a dB/dT value for the rTMS stimulation. Presumably a biphasic sine wave was used?

**Thank you for noting this important aspect in neuromodulation studies.**

**We used the Cool-40 Rat Coil (Parthoens, J., Verhaeghe, J., Servaes, S., Miranda, A., Stroobants, S., Staelens, S., 2016. Performance Characterization of an Actively Cooled Repetitive Transcranial Magnetic Stimulation Coil for the Rat. Neuromodulation: Technology at the Neural Interface 19, 459–468.** [**https://doi.org/10.1111/ner.12387**](https://doi.org/10.1111/ner.12387)**)**

**The coil has a self induction of 9,0, a max. di/dt 200A/µsec at 100% amplitude, and a pulse width of 262µsec. The maximum stimulation output used was 32%. As described in the paper, and the literature on performance of coils, the di/dt follows a linear response, and therefore the di/dt is 64A/µsec at 32%**

**Alekseichuk and colleagues characterized the e-field modelling of the Cool-40 coil estimating the absolute value of Emax at 180 mV/mm. (Alekseichuk, I., Mantell, K., Shirinpour, S., Opitz, A., 2019. Comparative modeling of transcranial magnetic and electric stimulation in mouse, monkey, and human. Neuroimage 194, 136–148.** [**https://doi.org/10.1016/j.neuroimage.2019.03.044**](https://doi.org/10.1016/j.neuroimage.2019.03.044)**).**

 **Supplemental figure S5 from the above referenced paper.**

**We have added this information to the methods section of the paper.**

- please check whether the duration listed (189 seconds) for iTBS is correct.

**Good question. We looked into this and indeed it is correct. The reason why the duration appears different from what one would calculate based on the stated train durations etc is because each cycle is not exactly 10 sec as we stated but in fact is 9.82 sec.**

- Was there a rationale for delivering rTMS every second day? rTMS is most commonly given daily for a period. An alternative protocol is fine to use, but a description of how that rationale was chosen would be useful.

**Thank you for pointing out this difference between the schedule we use and the most frequent use in clinical practice.**

**We are reporting in this paper secondary analyses of a set of experiments where we wanted to understand cellular and mollecular mechanisms of action of both ECS and TMS. While ECT is delivered twice or thrice a week (never on consecutive days), rTMS is most often delivered daily. We opted for harmonizing the protocols for every other day, considering the above and the fact that rTMS has been also delivered in clinical settings every other day and showing similar outcomes to daily treatments (Galletly, C., Gill, S., Clarke, P., Burton, C., Fitzgerald, P.B., 2012. A randomized trial comparing repetitive transcranial magnetic stimulation given 3 days/week and 5 days/week for the treatment of major depression: is efficacy related to the duration of treatment or the number of treatments? Psychol. Med. 42, 981–988.** [**https://doi.org/10.1017/S0033291711001760**](https://doi.org/10.1017/S0033291711001760)**).**

**Nonetheless, the above is included in the manuscript for clarity.**

- please provide the NA for the objectives used.

**These details have been added.**

- was there a reason 1-6 cells was counted per animal for dendritic spines? Counting 1 neuron vs 6 neurons is likely to have a large impact on the interpretation of the results.

**Good question. The main reason is simply because there are variable numbers of newborn neurons in the striatum of adult mice. For this reason, we did not calculate animal-level averages, but instead include data from each neuron in the dataset. By using mixed-effects models we can therefore effectively include all of the neuronal data while at the same time account for variation that may arise from animal-level differences.**

Discussion:
- It would be helpful to discuss the impact of the following on the results and their interpretation.

(1) the use of an rTMS coil that stimulates the entire brain and at least some of the spinal cord if not most of the peripheral nerves. This coil is known to activate neurons in the spinal cord of rats, and would therefore be reasonable to assume is not focal at all for the mouse brain.

**Based on e-field modelling work shared above, it would appear the it is rather unlikely to have suprathreshold effects outside of the brain (see S5 image above).**

(2) the use of a non-standard rTMS protocol. Potentially a lack of effect could be due to the spacing of the stimulation. While the results are interesting, noting that other rTMS protocols could still have potential is worth noting.

**Agreed – this is a good point. In the revised manuscript we have raised this issue in the discussion.**

Reviewer #2: The ms by Gaertner and colleagues describes the effects of ECT and rTMS on the survival and spine density of adult-born striatal neurons in mice. Both techniques did not alter striatal neurons survival and structural plasticity with the adopted stimulation patterns, which still needs to be pointed out.

Though of interest, the ms suffers from an unclear vocabulary, thus needing a strong language revision.

I have some major and few minor concerns.

Major points:
Introduction: Recent literature of rTMS effects on hippocampus, mPFC and M1 structural plasticity reported a stimulation-dependent modulation in mice; high-frequency rTMS increased dendritic plasticity in prefrontal and motor cortex, while 1 Hz stimulation induced structural plasticity in mature granule cells, as also dendritic complexity of newly generated neurons, though not increasing DG progenitor cells proliferation. These data shuld be discussed.

**These are good points – we have some discussion about different effects of stimulation patterns on neural plasticity.**

Methods: animals should be be better described in the initial paragraph.

**Thanks for this advice. We have re-read the first paragraph of the methods to check for clarity.**

Methods: Why did the average motor threshold was determined with a separate group of animals?

**This was simply to avoid having to individually determine the motor threshold for each mouse on each day, since increased the time required to treat the mice would add unnecessary stress since mice had to be restrained.**

Results: better to specify what the markers RFP, DARPP-32 and NeuN are used for.

**This has been added.**

Minor points:
Introduction: The sentence "rTMS and electroconvulsive shock (ECS), the animal model of ECT,..." is not clear at all. Please rephrase.

**We have adjusted this sentence for clarity.**

Introduction: The sentence "More recent studies indicate that neurogenesis in the ventral striatum is increased in response to chronic pain" needs more details.

**We agree it would be nice to include more information about this interesting study but our main point was to highlight several studies that have shown evidence for adult neurogenesis in the striatum, which is otherwise a fairly rarely described phenomenon. This study otherwise has relatively little relationship to our study so we felt it would be distracting to add too many other details from it to the introduction.**

Methods: The sentence "Sham-stimulated mice were restrained and treated identically with the exception that the stimulation intensity" is not at all clear!! Please rephrase.

**This sentence has been rewritten.**

Methods: specify the microscope and objective used for the acquisition of images

**This has been changed.**

Figure legend 3: modify "Each colored circle" with "each dot".

Reviewer #3: In their manuscript, Gaertner et al have investigated the effects of electroconvulsive shock (ECT) and 10Hz repetitive transcranial magnetic stimulation (rTMS) on the survival of adult born striatal neurons in the mouse brain. The author’s have used a transgenic mouse line to label adult-born neurons via tamoxifen injection, and beginning from 8 days later, applied ECT or iTBS rTMS for 10 sessions over 3 weeks. Neither ECT or rTMS induced changes in the amount of surviving adult-born striatal neurons or the density of dendritic spines. The manuscript is written well the findings are of interest to the field. My comments are detailed below:

Major comments
-The authors describe that they administered rTMS using a Cool-40 coil at 115% of motor threshold. Can the authors please provide detail on the estimated magnetic field or electric field at the surface of the cortex in their model. Additionally, there is evidence to suggest that this coil may lack focality and stimulate regions of the brain and spinal cord outside of the targeted region (<https://doi.org/10.1111/ner.12387>). Some acknowledgements of the non-focality of this coil should be included in the methods or discussion.

**Thank you for the comment. We were not able to find definite evidence that Cool-40 coil stimulates areas of the spinal cord.**

**The paper referenced above does not substantiate such claims but rather raises the question based on the lack of difference in MEP latency in a set of experiments the authors do not provide data for or details as to how they conducted such experiment (Parthoens, J., Verhaeghe, J., Servaes, S., Miranda, A., Stroobants, S., Staelens, S., 2016. Performance Characterization of an Actively Cooled Repetitive Transcranial Magnetic Stimulation Coil for the Rat. Neuromodulation: Technology at the Neural Interface 19, 459–468.** [**https://doi.org/10.1111/ner.12387**](https://doi.org/10.1111/ner.12387)**)**

**Page 465:**

**“*Focality of the Electric Field Distribution***

***The induced E-field in the spherical head model has a ringshaped distribution as was expected for a circular TMS coil (17) and together with the 40 mm outer coil diameter suggests a rather unfocal stimulation of the rat cerebral cortex. This was confirmed by the lack of laterality in the MT determination. The average latencies of the hind limb MEPs in the present study (8.23 6 0.14 msec and 8.38 6 0.16 msec) were comparable to those previously described after more focal stimulation of the rat’s motor cortex using a figure-of-eight coil (8.76 6 0.29 msec) (27). However, using our described coil we found comparable latencies when placing the coil directly onto the spinal cord of a propofol-anesthetized rat (data not shown). This lack of focality and lack of differences in the latency times raises the question whether the evoked potentials measured in the rat’s limbs in studies using a circular coil might be, solely or in part, caused by stimulation of regions further down the corticospinal tract.*”**

**The author’s speculation does not seem supported by modeling results by Alekseichuk and colleagues three years later in 2019 (Alekseichuk, I., Mantell, K., Shirinpour, S., Opitz, A., 2019. Comparative modeling of transcranial magnetic and electric stimulation in mouse, monkey, and human. Neuroimage 194, 136–148.** [**https://doi.org/10.1016/j.neuroimage.2019.03.044**](https://doi.org/10.1016/j.neuroimage.2019.03.044)**).**

-Were animals that were delivered ECT anaesthetised for the duration of the stimulation or was this conducted in non-anesthetised animals? If it was conducted in anesthetised animals, can details of the anaesthesia protocol please be detailed.

**Good catch. Animals were anesthetized for the duration of the stimulation. We have clarified this in the methods.**

-Can the authors please provide information on why the stimulation protocol was chosen. Why was stimulation delivered every second day? Also, why was tissue collected 3 days after the final stimulation day? Some discussion of how this stimulation protocol compares to other stimulation protocols and how the timing of tissue processing may have contributed to the results should be included.

**See our response to a similar question about the stimulation protocol from Reviewer #1, which have addressed through changes to the text. We have also added a statement to the discussion which notes that neurostimulation could induce plasticity in new neurons at timepoints other than the ones we investigated.**

-The discussion does not discuss the implications of the results and how these findings contribute to our current understanding of brain stimulation. Others studies using rTMS have demonstrated that it can modulate subcortical areas (<https://doi.org/10.1016/j.crneur.2022.100033>) and alter neurogenesis (ref 9 in the manuscript). Some discussion on how the current study fits in with these previous studies and its significance should be expanded on.

**To the best of our knowledge our work is the first to compare and contrast the effects of ECS and rTMS side by side. Moreover, while many studies have investigated hippocampal neurogenesis, there is only one study of ECS effects on striatal neurogenesis, and no studies of TMS effects on striatal neurogenesis. Ourt study therefore offers translational insights about the different mechanism of action of these two modalities. In the revised manuscript we have added text at the beginning of the discussion to emphasize the unique aspects of our study.**

-It is interesting that the authors chose to include both male and female animals in their design. Noting the small sample size when subdividing the treatment groups by sex, the authors may want to include some quantitative details of this output (e.g. a table showing differences between sex in their treatment groups) as there seems to be a small difference between sex in Fig 2v iTBS which may be important to note for future studies. Nonetheless, some acknowledgement on the effects of sex on their results should be included in the discussion.

**This is a good point. We didn’t want to draw too much attention to sex given that we were underpowered to perform a proper analysis, but the reviewer indeed noticed a more than twofold difference between ECS-treated males and sham males. We have noted this in the results and also mention it in the discussion in light of the greated prevalence of PD in men than in women.**

Minor comments
-The reference list seems to be duplicated and also slightly different between both lists. This should be amended.

**Thanks for catching this; the duplicate reference list has been deleted.**

-A reference should be supplied for the Allen mouse brain atlas (page 4).

**This has been added.**

-The enlarged images in the figures do not contain a scale bar (e.g. 1c, 1e, 2b-e, etc). Can a scale bar for these higher magnification images please be included.

**This has been added.**

-The y-axis for the bar graphs in the included figures should be a consistent scale (e.g. fig 2v).

**This has been corrected.**
